# Photoperiodically driven transcriptome-wide changes in the hypothalamus reveal transcriptional differences between physiologically contrasting seasonal life-history states in migratory songbirds

**DOI:** 10.1038/s41598-021-91951-4

**Published:** 2021-06-17

**Authors:** Aakansha Sharma, Subhajit Das, Sayantan Sur, Jyoti Tiwari, Khushboo Chaturvedi, Neha Agarwal, Shalie Malik, Sangeeta Rani, Vinod Kumar

**Affiliations:** 1 Department of Zoology, University of Delhi, Delhi, 110007 India; 2 Department of Zoology, University of Lucknow, Lucknow, 226007 India

**Keywords:** Animal physiology, Animal migration

## Abstract

We investigated time course of photoperiodically driven transcriptional responses in physiologically contrasting seasonal life-history states in migratory blackheaded buntings. Birds exhibiting unstimulated winter phenotype (photosensitive state; responsive to photostimulation) under 6-h short days, and regressed summer phenotype (photorefractory state; unresponsiveness to photostimulation) under 16-h long days, were released into an extended light period up to 22 h of the day. Increased *tshβ* and *dio2*, and decreased *dio3* mRNA levels in hypothalamus, and low *prdx4* and high *il1β* mRNA levels in blood confirmed photoperiodic induction by hour 18 in photosensitive birds. Further, at hours 10, 14, 18 and 22 of light exposure, the comparison of hypothalamus RNA-Seq results revealed transcriptional differences within and between states. Particularly, we found reduced expression at hour 14 of *transthyretin* and *proopiomelanocortin receptor*, and increased expression at hour 18 of *apolipoprotein A1* and carbon metabolism related genes in the photosensitive state. Similarly, valine, leucine and isoleucine degradation pathway genes and *superoxide dismutase 1* were upregulated, and *cocaine- and amphetamine-regulated transcript* and *gastrin-releasing peptide* were downregulated in the photosensitive state. These results show life-history-dependent activation of hypothalamic molecular pathways involved in initiation and maintenance of key biological processes as early as on the first long day.

## Introduction

Birds, like many other seasonally breeding vertebrates, switch seasonally from one life-history state (LHS) to the other, for example, from non-breeding to breeding to the post-breeding state; this maximizes the overall metabolic and reproductive fitness. There are distinct differences in daily and seasonal responses between life-history states comprising the annual cycle, as governed by the interaction of the endogenous circadian (Latin: *circa* = approximately; *dian* = a day) and circannual (Latin: *circa* = approximately; *annual* = a year) timekeeping mechanisms with prevailing natural day length (= photoperiod)^1,2^. Several laboratory studies have demonstrated the photoperiodic control of daily and seasonal responses in both non-migratory and migratory songbirds, using behavioral and physiological markers^3–7^. For example, under short days, the latitudinal migrant birds maintain their non-migratory/non-breeding winter phenotype (small gonads and daytime activity), and they remain responsive to the stimulatory effects of long day; this is referred to as the photosensitive state. Under long days, on the other hand, these birds undergo a complete growth-involution cycle such as the gain–loss in body fattening and weight, and recrudescence-regression of gonads, and they become unresponsive to the stimulatory effects of long day; this is referred to as the photorefractory state^3,7^. These two states provide a contrasting continuum of the induction and cessation of physiological processes underlying seasonal life-history states of the annual cycle in a photoperiodic species.

During the last few years, there is some evidence suggesting transcriptome wide changes in both central and peripheral tissues in parallel with photoperiod-induced transition in seasonal phenotypes in avian migrants. In captive Swainson's thrushes (*Catharus ustulatus*), for example, Johnston et al.^8^ reported that genes related to neuronal plasticity such as the cell adhesion, proliferation and motility were higher in expressions in the ventral hypothalamus in migratory, compared to the non-migratory state, suggesting extensive changes in brain with seasonal transition from non-migratory to the migratory state. Likewise, there were 547 differentially expressed genes in the transcriptome profile of blood and pectoral muscle tissues of the dark-eyed junco (*Junco hyemalis*) between its migratory and sedentary populations^9^. These differentially expressed genes that enrich the ribosomal structure (associated with erythropoiesis) in blood, and the lipid transport and fatty acid catabolic processes in muscles (associated with migratory fuel) were higher in expression levels in migrant (*J. h. hyemalis*), compared to the resident (*J. h. carolinensis*) juncos^9^. Further, a study on blood transcriptome showed differential expression patterns of genes associated with hyperphagia, moluting, DNA replication and transcription between resident and migrant populations of partially migrant European blackbirds, *Turdus merula*^10^. Similarly, RNA-Seq of hypothalamus and liver transcripts indicated significant differences in molecular pathways such as the ATP binding and calcium ion transport between photoperiodically stimulated non-migratory and migratory states of blackheaded buntings, *Emberiza melanocephala*^11^.

Less is understood about the dynamics of photoperiodically driven changes at the global transcription level during crucial seasonal life-history states of a photoperiodic species. In a long-day breeding species, however, a response to increasing spring photoperiods is triggered when daily light period surpasses the threshold photoperiod, e.g. ≥  ~ 12 h light per day^1^. The photoperiodic response is rapid, dramatic and robust, as demonstrated by the first day release (FDR) model of photoperiodism in which photoperiodic induction is tracked at intermittent hours after the animal is released from a short photoperiod to an extended period of light exposure^12^. Few photoperiodic birds like Japanese quails (*Coturnix c. japonica*) and migratory blackheaded buntings (*Emberiza melanocephala*) have been shown to respond to such an FDR experimental paradigm. Quails and buntings released from non-inductive 6 h short photoperiod to an extended light period showed a significant rise in the plasma luteinizing hormone (LH, which is released from pars distalis of the pituitary gland and regulates gonadal recrudescence) levels by hour 23 and hour 18, respectively, on the first day of extended light exposure^13,14^. Rapid LH rise is due to photoperiodic activation of the hypothalamic mechanisms, as shown by increased gonadotropin releasing hormone (GnRH) secretion by hour 22.5 of the first day of light exposure (first long day) in quail hypothalamic explants^15^.

**Figure 1:**
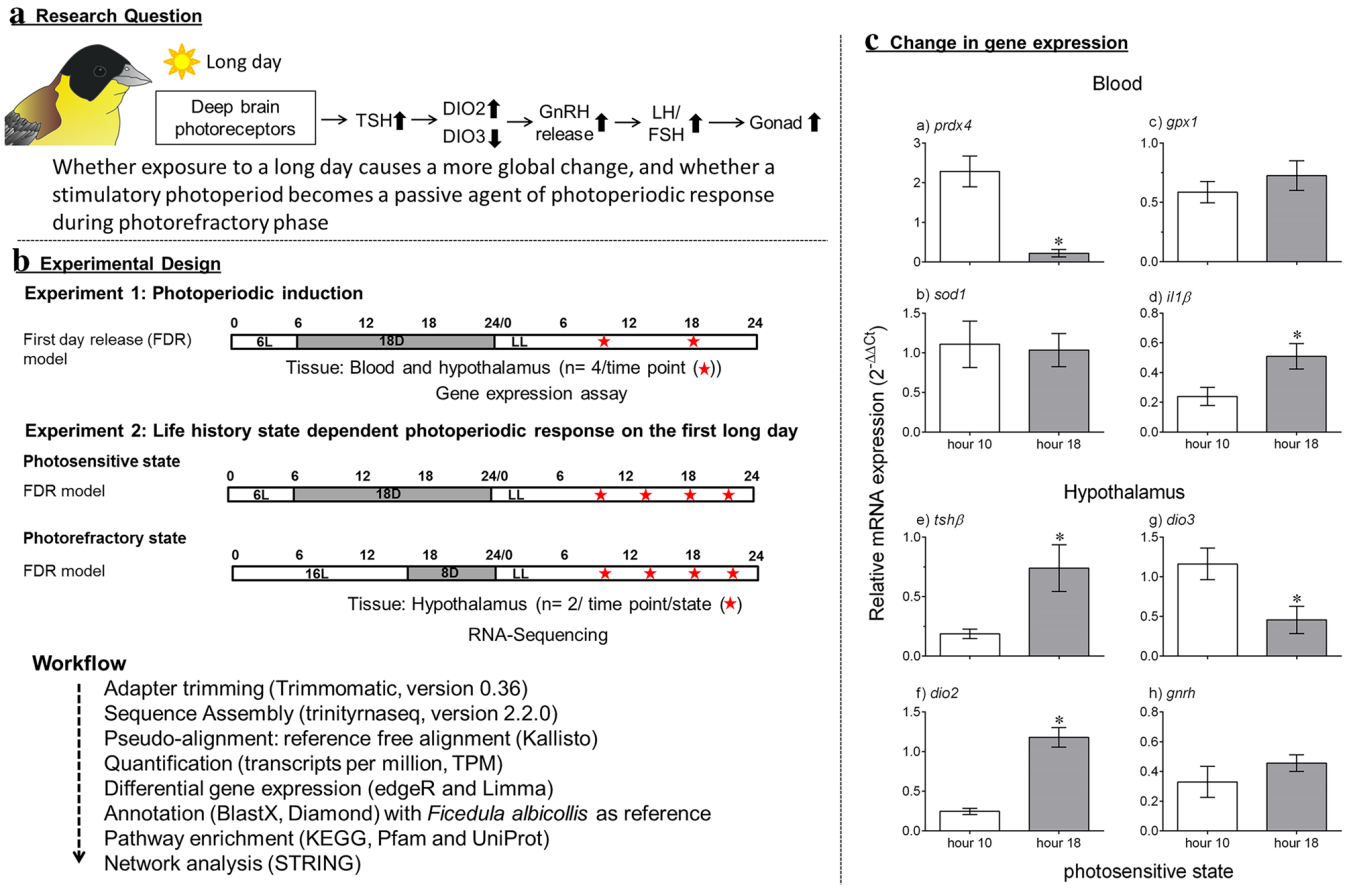
Left panel: (**a**) Research Question. (**b**) Shows the experiment design. In experiment 1, blackheaded buntings (*Emberiza melanocephala*; n = 8) were released from short photoperiods (6L:18D) into an extended light period, and sampled for blood and hypothalamus tissues at hours 10 and 18 (n = 4 per time point, hour 0 = light on) for gene expression assay to confirm the photoperiodic response. In experiment 2, photosensitive and photorefractory buntings (n = 8 each) maintained under 6L and 16L photoperiods, respectively, were released into an extended light period, and sampled for hypothalamus tissue at hours 10, 14, 18 and 22 (n = 2 per time point per group; hour 0 = light on) for RNA-Seq analysis. The sampling time points are indicated along the light period bars. A workflow for RNA-Seq analysis is also provided at the bottom. Right panel, (**c**) Mean (± SE, n = 4/time point) relative expression of genes in blood and hypothalamus of photosensitive blackheaded buntings at hours 10 and 18 on the first long day period (see above). a–d: Blood mRNA levels of *prdx4*, *sod1*, *gpx1* and *il1β* genes. e-h: Hypothalamic mRNA levels of thyroid hormone responsive genes involved in photoperiodic induction (*tshβ*, *dio2*, *dio3* and *gnrh*). An asterisk (*) on bar indicates a significant difference (Student’s unpaired t-test). For significance, alpha was set at 0.05.

The avian hypothalamus is the site for the perception and transduction of the photoperiod, the timekeeping mechanism(s) to assess the photoperiod change, and the effector (output) pathways that convert the photoperiodic message into a biological response^5,6,16^. The three component mechanisms involve the photoperiodic activation of a cascade of genes^5,16,17^. For instance, the neuropsin (Opn5) photopigment containing paraventricular hypothalamic neurons (deep brain photoreceptors) detect the light presence and, via G protein-coupled receptors, they convey the photoperiodic message to external zone of the median eminence, which is juxtaposed to pars tuberalis (PT) of the pituitary gland^18^. By hour 14 of the first long day, PT thyrotrophs show increased transcription of *tshβ*, which coupled with its alpha subunit (*tshα*) forms the thyroid stimulating hormone that binds to its receptors on the hypothalamic tanycytes (ependymal cells lining the third ventricle). By hour 18, there is the reciprocal switching of *dio2* and *dio3* gene expressions that code for type 2 and type 3 deiodinase enzymes, respectively^19^. Dio2 catalyzes the intracellular conversion of thyroxine (T4) to its biologically active form, the triiodothyronine (T3) that controls GnRH secretion into the hypophyseal portal system and consequently gonadal maturation in a seasonal species^20,21^ (Fig. 1a).

The transition from non-migratory to the migratory state may require changes associated with immune defense responses to cope with possible immunological challenges that avian migrants might face during the migratory travel^22^. This might involve majorly changes in the innate immune response, which is not energetically as expensive as the antibody production; these responses can be assessed by changes in mRNA expression of the cytokines, for example, *il1β* gene-encoded interleukin 1β^23^.

Increased lipogenesis (body fattening and weight gain) and nocturnal activity (akin to the migratory flight) in the migratory state of otherwise day active migrant songbird, can lead to an increased production of the reactive oxygen and nitrogen species (RONS), and an augmented oxidative stress^24,25^. Hence, birds need to activate their defense mechanism(s) against the oxidative stress in order to maintain the RONS balance during the period of activation of physiological processes associated with migratory preparedness. This can be assessed in blood by the measurement of genes that code for various antioxidant enzymes, for example, the peroxiredoxin 4, superoxide dismutase 1 and glutathione peroxidase 1 enzymes. Whereas, the *prdx4*-encoded peroxiredoxin 4 scavenges the hydrogen peroxides and plays a crucial role in cellular response to the oxidative stress and intracellular transduction^26^; the *sod1*-encoded superoxide dismutase 1 dismutates superoxide radicals to oxygen and hydrogen peroxide^27^, and *gpx1*-encoded glutathione peroxidase 1 reduces the hydrogen and lipid peroxides^28^.

The question is: (i) whether the daily photoperiod, arguably the most important input from environment, induces a more global molecular changes rather than its widely investigated effects on the reproductive axis, and (ii) whether a long day is a passive agent of seasonal change during the photorefractory state (Fig. 1a). We addressed these, using the FDR model of photoperiodism (see above; Fig. 1b) in migratory blackheaded buntings, which exhibit photoperiodically controlled seasonal LHSs, and show a rapid photoperiodic LH response by hour 18 on the first long day^14,29–31^. First, we confirmed the photoperiodic response at the molecular level in central (hypothalamus) and peripheral (blood) tissues in photosensitive blackheaded buntings that were released from 6 h short photoperiod to extended light period up to hour 22 of the day. Then, we assessed differences in hypothalamic transcriptional response of buntings between physiological contrasting photosensitive and photorefractory seasonal LHSs. The photosensitive LHS was characterized by the non-migratory/non-breeding winter phenotype (no body fattening, normal body mass, small reproductively immature testes, daytime activity, and responsiveness to long days) in birds maintained under short days. Similarly, the photorefractory LHS was characterized by the post-migratory/post-breeding summer phenotype (no body fattening, lean body mass, regressed gonads and return to daytime activity, and loss of responsiveness to long days) following the growth-involution cycle under long days. These two photoperiodic states with almost similar phenotypes represented physiologically contrasting LHSs of the blackheaded bunting’s annual cycle^29–31^. For RNA-Seq, we collected hypothalamus samples from both photosensitive and photorefractory states at hours 10, 14, 18 and 22, covering important time points of the photoinducible phase (the part of daily cycle that is reponsive to the photoperiodic induction) to show time-course of photoperiodic response on the first long day^1,13,14,19,32^. The prediction was that transcriptome-wide changes would reveal seasonal differences in the dynamics of hypothalamic molecular pathways that are triggered as early as within few hours of the exposure to light period surpassing the threshold photoperiod for the induction of the photoperiodic response in a seasonal species.

## Results and discussion

### A single long day induces the photoperiodic molecular response

Figure 1c shows results from the experiment 1, as evidenced from the qPCR measurement of mRNA expression of genes of known biological functions in the blood and hypothalamus. Clearly, the exposure to extended light period induced a molecular response by hour 18 of the first long day, as shown by change in mRNA levels of candidate genes in both central (hypothalamus) peripheral (blood) tissues of photosensitive buntings. Blood mRNA levels of *peroxiredoxin 4* (*prdx4*) were significantly lower at hour 18 mimicking a long 18 h photoperiod than those at hour 10 mimicking a short 10 h photoperiod (*p* = 0.002, t = 5.18, n = 4/time point). Paradoxically, this indicated a reduced cellular response against oxidative stress in the otherwise photo stimulated birds on the first long day. We speculate that *prdx4* expression pattern would be inversed (i.e. increased *prdx4* mRNA levels) after several long days when birds show photoperiodically stimulated hyperphagia (increased food intake) and lipogenesis (fat accumulation). Intriguingly, however, blood mRNA levels of *gpx1* (*p* = 0.399, t = 0.91, n = 4/time point) and *sod1* (*p* = 0.845, t = 0.20, n = 4/time point) genes were not different between hours 10 and 18 (Student’s t-test, Fig. 1c(a–c)). Taken together differences in the expression pattern of these enzymes, we speculate differential activation of the enzymatic pathways that are probably involved in the oxidative cellular response when migratory birds are exposed to an acute change in their photoperiodic environment.

On the other hand, blood *il1β* mRNA levels were significantly higher at hour 18 than the hour 10 (*p* = 0.041, t = 2.58, n = 4/time point; Student’s t-test, Fig. 1c(d)). It is consistent with the known role of *il1β*-encoded interleukin 1β, as a crucial mediator of the inflammation and a marker of the innate immune system^22,23^. Increased *il1β* mRNA expression on the first long day is consistent with the idea of parallel photoperiodic induction of multiple biological processes, including those associated with the innate immune response, body fattening and gonadal maturation in migratory songbirds^28^; however, the possibility that an upregulated interleukin was an indicative a stress response cannot be excluded at this time.

Changes in hypothalamic gene expressions further confirm a rapid molecular response to the extended light period when it surpasses the threshold photoperiod, i.e. acts as the stimulatory long day. Reciprocal switching of genes involved in the thyroid hormone responsive pathway at hour 18 particularly evidences this. Hypothalamic mRNA levels of *tshβ* (*p* = 0.033, t = 2.75, n = 4/time point) and *dio2* (*p* = 0.0004, t = 7.14, n = 4/time point) genes were higher, and that of *dio3* gene expression was lower at hour 18 than the hour 10 (*p* = 0.036, t = 2.68, n = 4/time point). This is also in agreement with the rapid photoperiodic response found on the first long day in plasma LH secretion, and in hypothalamic expressions of Fos-immunoreactivity and thyroid hormone responsive genes in blackheaded buntings^14,33^ and other photoperiodic birds^15,17,19,32,34–38^. However, *gnrh* mRNA levels were not found significantly different between hours 10 and 18 of the first long day (*p* = 0.324, t = 1.07, n = 4/time point; Student’s t-test, Fig. 1c(e–h) indicating that hour 18 was probably too early a time for an upregulated *gnrh* expression on the first long day^37–39^.

### RNA-Seq reveals differences in time course of the photoperiodic response

Table S2 summarizes the primary statistics used for RNA-Seq results. Using only transcripts with non-zero abundance, we compared the time course of transcriptome-wide response in the hypothalamus both as the function of time (within photosensitive or photorefractory state) and LHS (photosensitive vs. photorefractory state; n = 2/time point/state except at hour 22 in photorefractory state which had n = 1 sample size). Further, to show a functional linkage of differentially expressed genes (DEGs), we performed STRING analysis that predicts the protein–protein interaction (see methods for details).

Results on hypothalamic gene expressions suggest that buntings react to the acute photoperiodic change in photorefractory state almost as they do in the photosensitive state. However, the comparison of the overall RNASeq data from both states revealed LHS-dependent pattern in the time course of transcriptional response, with differences in the number and functions of DEGs and associated physiological pathways.

#### Within state differences in time course of transcriptional response

We examined the time course of response on the first long day, by comparing gene expressions at the hours 14, 18 and 22 of the extended light period that mimicked 14 h, 18 h and 22 h long photoperiods, respectively, with those at hour 10 that mimicked a 10 h short photoperiod.

##### Photosensitive state

**Figure 2:**
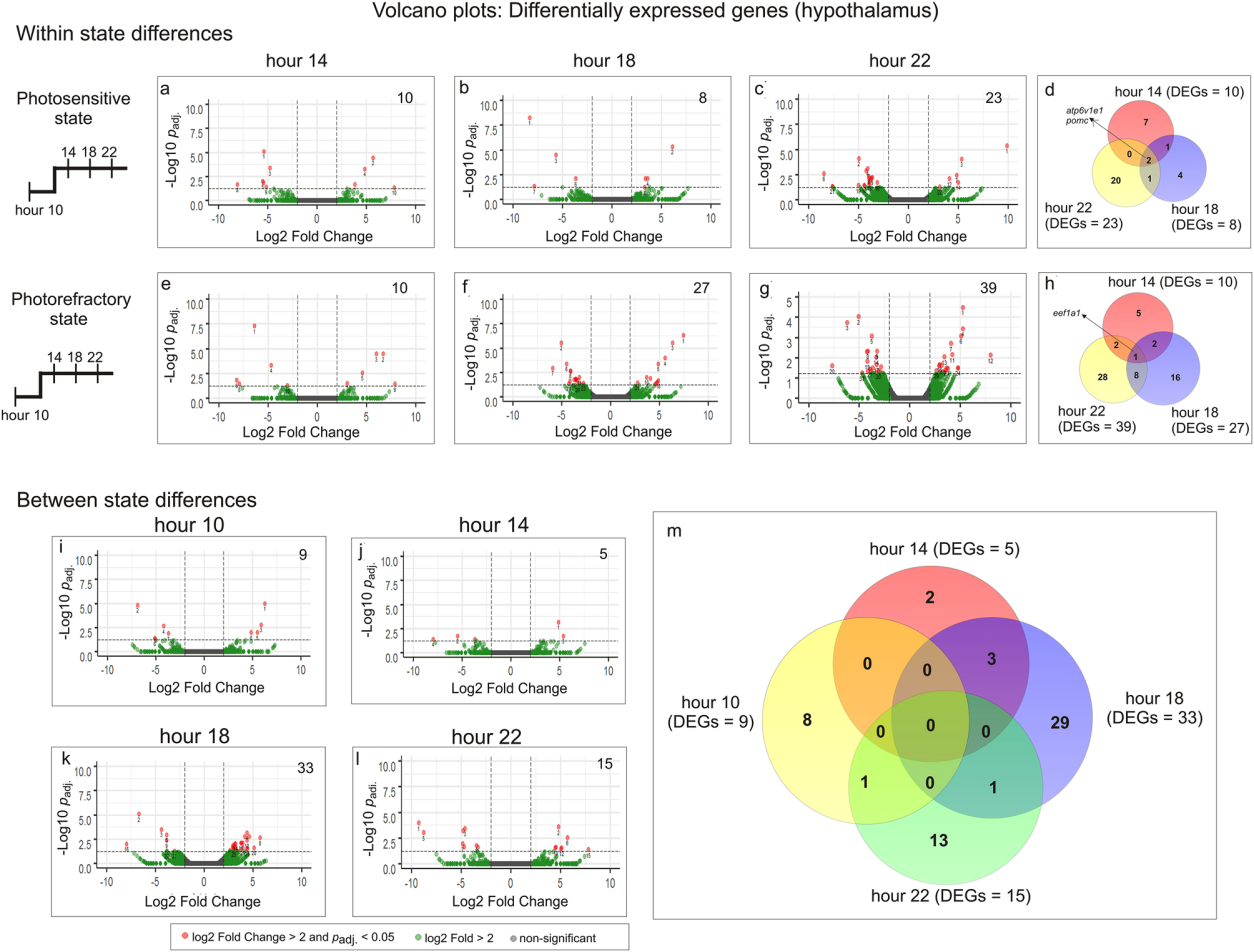
Top panel: Volcano plots showing results of differential gene expression analysis (− log10 padj. vs. log2 fold change values) in the hypothalamus within the photosensitive (**a**–**c**) and photorefractory states (**e**–**g**). The comparison protocol is shown on the left. In each state, the comparisons were done with respect to the hour 10 value (akin to short day control). Venn diagram shows common and unique DEGs in photosensitive (**d**) and photorefractory states (**h**). Bottom panel: Volcano plots showing results of differential gene expression analysis (− log10 padj. vs. log2 fold change values) between the photosensitive and photorefractory states. The pairwise comparisons were made at all the four time points (hours 10 (**i**), 14 (**j**), 18 (**k**) and 22 (**l**)). Venn diagram shows common and unique DEGs between states at hours 10, 14, 18 and 22 (**m**). Genes in a volcano plot with log2 fold change > 2 are marked by green colour, and those with log2 fold change > 2 and *p* value (*p*_adj._) < 0.05 are marked by the red dots.

**Figure 3:**
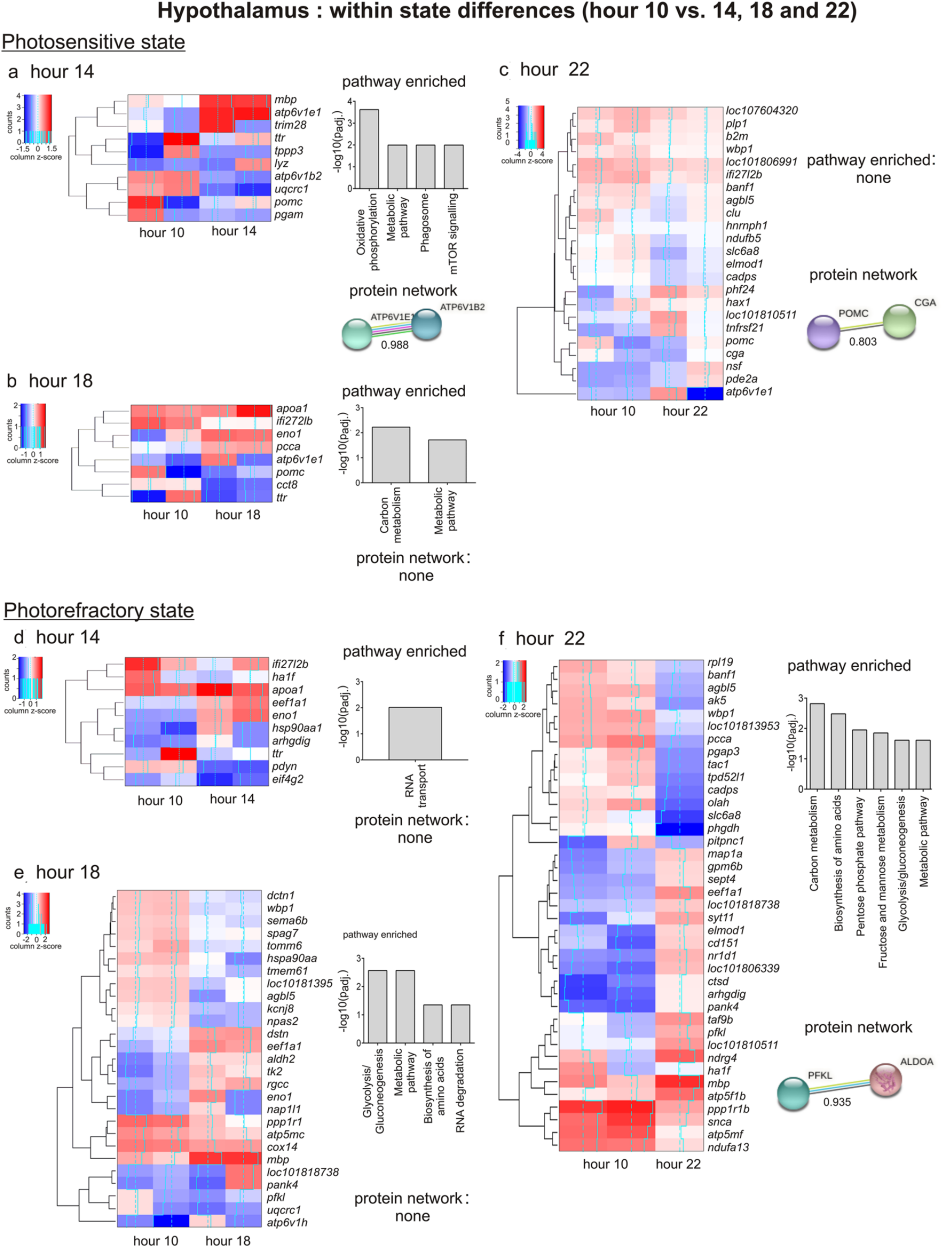
Heatmap of differentially expressed genes (DEGs) in the hypothalamus in each state (n = 2/time point/state except at hour 22 in photorefractory state where n = 1). DEGs (rows) were determined by comparing the expression value at hour 14, hour 18 or hour 22 with hour 10 value (column). Log-transformed values were normalized and plotted as z-scores (blue, minimum; red, maximum). A blue continuous line running through heatmap shows expression differences from mean value (dashed line). Genes with similar expression patterns have been clustered together. The right panel shows the results of significantly enriched pathways (*p*_adj._ < 0.05), and STRING DEG network analysis in which the nodes/circles represent gene-encoded proteins, while the edge/lines connecting circles represent the protein–protein interaction. The empty nodes represent proteins of an unknown 3D structure, while the filled nodes represent the known or a predicted 3D structure.

At hour 14, we found 10 differentially expressed genes (DEGs) with 4 upregulated and 6 downregulated genes (Figs. 2a, 3a, Table S3). Of the 10 DEGs, *atp6v1e1*, *atp6v1b2*, *uqcrc1* and *pgam1* genes enriched the oxidative phosphorylation, metabolic pathways, phagosome and mTOR signalling pathways (Table 1). The oxidative phosphorylation and metabolic pathways were upregulated at hour 10**,** while the phagosome and mTOR signalling pathways were enriched by two genes that were opposite in the expression trend: *atp6v1e1* was upregulated while *atp6v1b2* was downregulated at hour 14. The STRING analysis showed a significant interaction of *atp6v1e1* and *atp6v1b2* encoded proteins (ATP6V1E1 and ATP6V1B2). These proteins are the components of vacuolar ATPase enzyme that mediates the acidification of eukaryotic intracellular organelles necessary for protein sorting and zymogen activation. Further, at hour 14, *ttr* gene that codes for transthyretin (a preferential T3 binder) and *pomc* gene that codes for the proopiomelanocortin receptor had significantly lower expressions. Whereas, low *ttr* gene expression, as in photostimulated redheaded buntings^40^, might indicate a reduced trafficking of thyroid hormones via *ttr*-encoded transthyretins in the photosensitive state, the low *pomc* gene expression might suggest the removal of inhibitory effects of the opioids (e.g. β-endorphin, a *pomc*-encoded proopiomelanocortin product) on hypothalamic GnRH and, in turn, pituitary LH secretion^41,42^.

**Table 1:** Results of pathway enrichment of differentially expressed genes within and between state comparisons in the hypothalamus.

Category	Pathway#	Pathway description	Differentially expressed genes	P_adj_	Bias group
**A. Hypothalamus: within state comparison (*Photosensitive*: *hour 10 vs. hour 14*)**					
KEGG	fab00190	Oxidative phosphorylation	3	0.0002	Hour 10
KEGG	fab01100	Metabolic pathways	4	0.0102	Hour 10
KEGG	fab04145	Phagosome	2	0.0102	*–*
KEGG	fab04150	mTOR signaling pathway	2	0.0102	*–*
**(*Photosensitive*: *hour 10 vs. hour 18*)**					
KEGG	fab01200	Carbon metabolism	2	0.006	Hour 18
KEGG	fab01100	Metabolic pathways	3	0.019	Hour 18
**(*Photorefractory*: *hour 10 vs. hour 14*)**					
KEGG	fab03013	RNA transport	2	0.009	*–*
**(*Photorefractory*: *hour 10 vs. hour 18*)**					
KEGG	fab00010	Glycolysis/Gluconeogenesis	3	0.002	Hour 18
KEGG	fab01100	Metabolic pathways	8	0.002	Hour 18
KEGG	fab01230	Biosynthesis of amino acids	2	0.044	–
KEGG	fab03018	RNA degradation	2	0.044	–
**(*Photorefractory*: *hour 10 vs. hour 22*)**					
KEGG	fab01200	Carbon metabolism	4	0.001	–
KEGG	fab01230	Biosynthesis of amino acids	3	0.003	Hour 22
KEGG	fab00030	Pentose phosphate pathway	2	0.011	Hour 22
KEGG	fab00051	Fructose and mannose metabolism	2	0.014	Hour 22
KEGG	fab00010	Glycolysis/Gluconeogenesis	2	0.024	Hour 22
KEGG	fab01100	Metabolic pathways	7	0.024	Hour 10
**B. Hypothalamus: Between state comparisons (*hour 10*: *photosensitive vs. photorefractory*)**					
KEGG	fab00280	Valine, leucine and isoleucine degradation	2	0.009	Photosensitive
KEGG	fab01100	Metabolic pathways	4	0.027	Photosensitive
KEGG	fab04145	Phagosome	2	0.027	Photosensitive
**(*hour 22*: *photosensitive vs. photorefractory*)**					
UniProt	KW-0689	Ribosome	2	0.021	Photosensitive

The column on ‘bias group’ indicates the category with a higher number of upregulated genes.

The blanks (-) in ‘bias group’ column indicate that the number of up- and downregulated genes in that pathway were equal.

Likewise, there were 8 DEGs with 4 upregulated and 4 downregulated genes at hour 18 (Figs. 2b, 3b, Table S3), of which *pcca*, *eno1* and *atp6v1e1* genes enriched the upregulated carbon metabolism and other metabolic pathways (Table 1). Besides, both *ttr* and *pomc* genes were downregulated, while *apoA-1* gene coding for apolipoprotein-A1 was upregulated in expression at the hour 18. An increased *apoa1* gene expression probably indicated the activation of the cholesterol metabolism pathway, as *apoa1*-encoded apolipoprotein-A1 is a major cholesterol transporter protein in both central and peripheral tissues, and regulates energy homeostasis^43^. Increased brain *apoa1* gene expression has been linked also with the photostimulated development migratory phenotype in migratory northern wheatears, *Oenanthe oenanthe*^44^.

At hour 22, we found 23 DEGs with 6 upregulated and 17 downregulated genes (Figs. 2c, 3c, Table S3), although these did not enrich a functional pathway. Downregulated *cga* and *pomc* genes seem to be the part of a protein network, as revealed by STRING analysis showing the network interaction of *cga* and *pomc* encoded proteins (CGA and POMC). It may be recalled that *cga* is alpha subunit of *tsh* gene (*tshα*) couples with its beta subunit (*tshβ*) and forms the thyroid stimulating hormone in pars tuberalis, which is part of the local thyroid hormone pathway that mediates the induction of a photoperiodic response^19,20,32^.

The *atp6v1e1* and *pomc* genes were differentially expressed at hours 14, 18 and 22 during the extended 22 h of the light period (Fig. 2d, Table S4). Whereas, as compared to their expressions at hour 10 mimicking a short day, *atp6v1e1* gene was upregulated, the *pomc* gene was downregulated in expression at all these three time points although they all mimicked a stimulatory long day (Figs. 2d; 3a–c).

##### Photorefractory state

There were transcriptional changes across the second half of the day, as indicated by the comparison of gene expression patterns at hours 14, 18 and 22 (long day), with that at hour 10 (short day). At hour 14, for example, we found 10 DEGs with 5 each having upregulated and downregulated expressions (Figs. 2e, 3d, Table S3). Differentially expressed *eif4g2* and *eef1a1* genes significantly enriched the “RNA transport” pathway, suggesting a translational effect of the light exposure at hour 14. Consistent with an upregulated translation activity at hour 14, the expressions of *eif4g2* and *eef1a1* genes were downregulated and upregulated, respectively (Fig. 3d, Table 1). Whereas *eef1a1* is a translational elongation factor gene, *eif4g2* gene acts as a general repressor of the translation; hence both these genes possibly interact and regulate translational processes following the photoperiodic induction of a response.

Likewise, there were a total of 27 DEGs with 11 upregulated and 16 downregulated genes at hour 18, Figs. 2f, 3e, Table S3), of which 8 DEGs enriched 4 functional pathways, namely the glycolysis/gluconeogenesis, metabolism, biosynthesis of amino acids and RNA degradation pathways (Fig. 3e, Table 1). In particular, we found an upregulated expression of genes associated with glycolysis/gluconeogenesis and metabolic pathways. Further, the upregulated *eno1* gene and downregulated *pfkl* gene enriched the amino acids biosynthesis and RNA degradation pathways. Differentially expressed *npas2* gene could be indicative of differences in in the circadian timing mechanism, although it remains purely speculative in the absence of differential expression of other circadian genes in the current study.

Similarly, at hour 22, we found 39 DEGs with 19 upregulated and 20 downregulated genes (Figs. 2g, 3f, Table S3). Here, 7 DEGs enriched six pathways of which genes including the biosynthesis of amino acids, pentose phosphate pathway, fructose and mannose metabolism, and glycolysis/gluconeogenesis were highly expressed at hour 22. At the same time, genes that enriched the metabolic pathway were lower in expression at hour 22 than the hour 10. The STRING analysis showed an interaction of *pfkl* and *aldoa* encoded glycolytic enzyme proteins (Fig. 3f)*.*

The *eef1a1* gene was also found to have an upregulated expression at all three timepoints (hours 14, 18 and 22), compared to the hour 10 of the first long day (Fig. 2h, Table S4). This though suggests daily changes in the translational processes in photorefractory birds, although these were probably were not as robust as found in photosensitive birds in which there was also concurrent downregulated expression of the *eif4g2* gene, a general repressor of the translation.

#### LHS-dependent time course of transcriptional response

**Figure 4:**
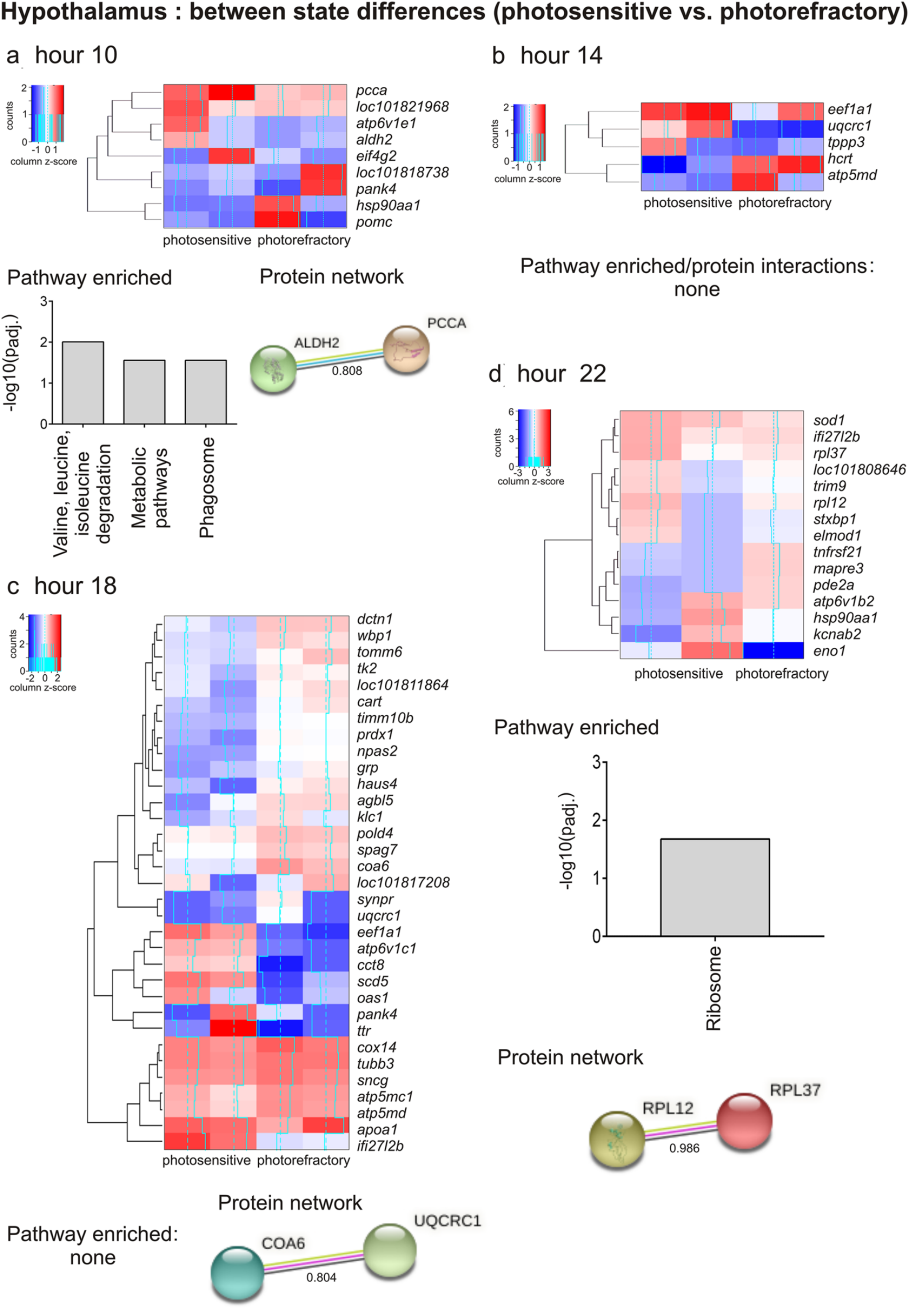
Heatmap of differentially expressed genes (DEGs) in the hypothalamus of bunting exhibiting photosensitive and photorefractory states (rows) at hours 10 (**a**), hour 14 (**b**) and hour 18 (**c**) and hour 22 (column) (**d**) (n = 2/time point/state except at hour 22 in the photorefractory state in which n = 1). Log-transformed values were normalized and plotted as z-scores (blue, minimum; red, maximum). A blue continuous line running through the heatmap shows expression differences from mean value (dashed line). Genes with similar expression patterns have been clustered together. Underneath each heatmap, the results of significantly enriched pathways (*p*_adj._ < 0.05), and STRING DEG network analysis in which the nodes/circles represent gene-encoded proteins, while the edge/lines connecting circles represent the protein–protein interactions. The empty nodes represent proteins of an unknown 3D structure, while the filled nodes represent the known or a predicted 3D structure.

Differences in the transcriptome-wide response between physiologically contrasting photosensitive and photorefractory states suggest that the time course of transcriptional activation of molecular processes differed between seasonal LHSs comprising annual cycle of migratory buntings. This is shown by differential gene expressions between two states at four times of the day that we have examined and compared in the current study. To begin with, at hour 10, we found 9 DEGs with 4 upregulated and 5 downregulated genes in photorefractory, compared to the photosensitive state (Figs. 2i, 4a; Table S3). Five DEGs enriched three functional pathways (valine, leucine and isoleucine degradation, metabolic, and phagosome pathways) that were upregulated in the photosensitive state (Fig. 4a, Table 1). The amino acids valine, leucine and isoleucine are involved in the biosynthesis of glutamate, which as a major excitatory neurotransmitter is involved in transmission of the photoperiodic information, and serves as a precursor molecule for the biosynthesis of largely inhibitory gamma aminobutyric acid (GABA) neurotransmitter^45^. The upregulated expression of genes associated with these pathways suggested an enhanced neural activity in photosensitive birds in response to their exposure to a stimulatory long light period. This is consistent with evidences for increased neural activity and neurogenesis in the hippocampus and nidopallium caudolaterale brain regions of photostimulated migratory white crowned sparrows, *Zonotrichia leucophrys*^46^ and reed warblers, *Acrocephalus scirpaceus*^47^). An increased neuronal activity, as shown by Fos-like immunoreactivity, has also been found in the mediobasal hypothalamus brain region of Japanese quails^17,34^ and blackheaded buntings^33^ in response to the first long day.

At hour 14, we found 5 DEGs with 2 upregulated and 3 downregulated genes in photorefractory, compared to the photosensitive state, although these DEGs did not significantly enrich a particular functional pathway (Figs. 2j, 4b, Table S3). Intriguingly, we also found a significantly upregulated expression of orexin precursor *hcrt* gene in the photorefractory state, which probably indicates an association of *hcrt* expression with LHS-dependent differences in sleep–wake pattern in buntings as they were exposed for many weeks to the 16 h long photoperiod. It is reported that *hcrt*-encoded hypocretin produced by lateral hypothalamus is essential for the arousal stability^48^, and activated hypocretin-producing neurons by direct photostimulation have been found associated with transition from sleep to awake state in mice^49^.

Similarly, at hour 18, 33 DEGs with 25 upregulated and 8 downregulated genes in photorefractory, compared to the photosensitive state, did not enrich a functional pathway (Figs. 2k, 4c, Table S3). Particularly, we found an upregulated *ttr*, and downregulated *cart* (coding for cocaine- and amphetamine-regulated transcript) and *grp* (coding for gastrin releasing peptide) expressions in photosensitive, compared to photorefractory birds, consistent with photoperiodic initiation of physiological processes. Whereas increased *ttr* gene expression might indicate enhanced activity of the photostimulated thyroid hormone responsive pathway, the decreased *cart* and *grp* gene expressions probably suggest the activation of processes associated with the hyperphagia and consequently the body fattening and weight gain in photosensitive birds exposed to stimulatory long days^50–52^. Further, there seems to be changes in genes associated with metabolic processes, as suggested by downregulated expression of *coa6* and *uqcrc1* genes in the photosensitive state. These genes are part of the respiratory chain complexes, and seem to interact as suggested from significant interaction of proteins that they encode for (Fig. 4c; STRING analysis).

At hour 22, we found 15 DEGs with 7 upregulated and 8 downregulated genes in photorefractory, as compared to the photosensitive state (Figs. 2l, 4d, Table S3). Of these DEGs, *rpl12* and *rpl37* genes that significantly enrich the ribosome pathway (Fig. 4d, Table 1) were upregulated in the photosensitive, as compared to expression in the photorefractory state (Table 1). This suggests the initiation of translational processes later in the night (hour 22) when birds were exposed to light. At this time, an upregulated *sod1* expression also suggests the activation of oxidative stress pathways pathway in photosensitive birds, as has been reported recently in photostimulated redheaded buntings^28^. We did not find a common gene that was differentially expressed at all the four time points between the photosensitive and photorefractory states (Fig. 2m; Table S4).

The exposure to a single long day activated a series of hypothalamic molecular pathways that are crucial for the initiation and maintenance of key biological processes during a seasonal LHS. We interpret that within and between state differences in the time course of transcriptional responses were due to differential light-sensitivity of the underlying circadian photoperiod-responsive rhythm, which is shown to be involved in regulation of the lipogenesis (body fattening and weight gain) and gametogenesis (gonadal maturation) in migratory blackheaded buntings and other birds in response to the long photoperiod^1,3,38,53^. Clearly, the photoperiodic induction depends on the extension of the light into the photoinducible phase lying in the second half (about 12 h after the light onset) of the endogenous circadian photoperiodic rhythm^1,12,54^. Present experiments also evidence this, by a much smaller transcriptional response at hour 10. There can also be LHS-dependent alteration in the 24-h waveform of the circadian rhythm governing the photoperiod-induced response in blackheaded buntings^31,55^. Differential responsiveness of the circadian photoperiodic rhythm seems to be the part of the overall adaptive strategy in avian migrants, which need to begin and end their spring migration to restrict their reproduction during most profitable time of the year. This, we believe, is achieved by the interaction of circadian rhythm and photoperiodism, which are mutually inclusive physiological processes, in a long day species like the migratory blackheaded bunting.

Present results should be viewed with a caution, however. This is because we have not shown changes in known marker genes of the photoperiodic response (e.g. *tshβ*, *dio2* and *dio3*) in the present RNA-seq study. This is intriguing, but we speculate that such an unexpected discrepancy was because of two possibly. First, the quality filtration used for hypothalamus RNA-Seq led to the loss from the list of data on ‘photoperiodic genes’, which are expressed in low amounts and site-specific manner^19,20^. Secondly, in the absence of full gene sequence of our study species (*Emberiza melanocephala*; family – Emberizidae), we used the reference genome of migratory *Ficedula albicollis* (family—Muscicapidae), and this probably has filtered out many functionally relevant genes from the annotation of present transcriptome. Despite these limitations of the current study, the striking differences in time course of the transcriptome-wide response both within the state and between states provide a strong evidence for LHS-dependence of the photoperiodically driven activation of hypothalamic molecular pathways during the annual cycle in migratory songbirds.

## Materials and methods

### Animal maintenance and experiment

The study was approved by the Institutional Animal Ethics Committee of the Department of Zoology, University of Lucknow, India (protocol # LU/ZOOL/IAEC/5/16/01/2A). All the experiments were performed in accordance with the Institutional guidelines and regulations. The study was in compliance with the ARRIVE guidelines.

The experiment used adult male blackheaded buntings (*Emberiza melanocephala*) captured from the overwintering flock, and acclimated to semi-natural (captive) conditions for a week in the outdoor aviary (size = 3 × 2.5 × 2.5 m) before being used in the experiment. Acclimated birds brought indoors and exposed at constant temperature (22 ± 2 °C) to short days (SD: 8 h light: 16 h darkness, 8L:16D) or long days (LD: 16L:8D) for the next 40 weeks. Under SD, blackheaded buntings maintain unstimulated photosensitive state; these birds have normal body mass and small reproductively immature gonads, and are responsive to stimulatory effects of long photoperiods, as during the late winter and early spring. Under LD, following growth-regression cycles, buntings have lean body mass and regressed reproductively inactive gonads, and cease their responsiveness (become photorefractory) to stimulatory effects of long photoperiods, as during post reproductive period of summer and early autumn^29–31,56,57^. Thus, regardless of crucial differences in the seasonal LHSs, blackheaded buntings seemed to have similar body mass and testes size at the beginning of the experiment^29–31,56,57^.

Using an identical experimental design, we performed two experiments to address the research questions that we had formulated for this study (Fig. 1a,b). Experiment 1 used photosensitive birds (body mass = 24.0 ± 1.0 g; n = 8) and confirmed the photoperiodic induction on the first day of extended light period (LD), as assessed by changes in mRNA expression of genes of known functions in blood and hypothalamus tissues. Birds were singly housed in activity cages (size = 18 × 10 × 20 cm) and kept in separate photoperiodic boxes (size = 24 × 15 × 30 cm; 1 bird/cage/box). To these birds, the light-on period for next 2 weeks was shortened to 6 h by advancing the light-off time (6L:18D); this was done to enhance buntings’ responsiveness to the stimulatory effects of single long day^58^. On day 15, the light-off time was removed from the timer, and 4 birds each at hour 10 and 18 of the extended light period were sacrificed, and blood and brain samples were collected. This is called the FDR paradigm, which has been used previously to show a rapid photoperiodic LH response in blackheaded buntings^14^.

Experiment 2 had an identical experimental design and used blackheaded buntings that were photoperiodically induced in both photosensitive and photorefractory state (see above). It examined whether response to light later in the day in a FDR paradigm was a more global change at the transcriptional level. This experiment also tested if long days were a passive agent of the photoperiodism during the post-breeding refractory LHS, unlike the pre-breeding photosensitive LHS of the annual cycle in migratory blackheaded buntings. We, therefore, compared the time course response of photosensitive with photorefractory buntings on the first day of an extended light period (n = 8 each state; body mass = 24.0 ± 1.0 g). As in experiment 1, birds were singly housed in separate photoperiodic boxes (1 bird/cage/box), and for next 2 weeks, photosensitive birds were maintained on 6L:18D, while photorefractory birds remained on 16L:8D, as before; the light-on times were identical for all birds. On day 15, the light-off time was removed from the timer, and beginning at hour 10, brain from 2 birds of each state was collected at 4-h intervals during the light on period, i.e. at hours 10, 14, 18 and 22 (hour 0 = time of lights on; n = 2/time point/group; Fig. 1b).

For collecting tissue samples, birds were decapitated to preclude possible anaesthesia effects on mRNA expression of genes^59,60^. Decapitation is a quick, unanticipated procedure lasting for < 10 s. We collected blood, and quickly excised out the hypothalamus from brain^55^; the latter was placed in the RNA-later at 4 °C overnight. Both blood and hypothalamus samples were then kept at − 80 °C until processed further.

### Gene expression assays

In experiment 1, we measured mRNA expression of 4 genes each in blood (*prdx4*, *sod1*, *gpx1* encoding antioxidant enzymes, and *il1*β encoding a cytokines) and hypothalamus (thyroid hormone responsive pathway genes, *tshβ*, *dio2*, *dio3* and *gnrh*) by quantitative PCR (qPCR), as standardized and routinely used in our laboratory^11,57^. For each gene assay, we used n = 4 samples of blood and hypothalamus.

For this, total RNA from each blood and hypothalamus sample was extracted using Trizol reagent (AM9738; Ambion, Austin, TX, USA), according to the manufacturer’s protocol, and as standardized and routinely used in our laboratory^11,57^. We used gene-specific primers, as published earlier from our laboratory^37,38^ (Table S1), and performed qPCR using SYBR green chemistry (Applied Biosystems, Life Technology, 4,367,659) run on ViiA7 realtime PCR system (Applied Biosystems, Foster City, CA, USA). Both sample and reference genes (*b-actin*) were run in duplicates, and the relative mRNA expression levels were determined as 2^−ΔΔCt^ ^61^. We ran qPCR for 40 cycles, each lasting for 75 s (melting at 95 °C for 15 s and annealing at 60 °C for 60 s), with *beta-actin* used as the reference gene which was found to be the most stable among potential reference genes (*beta-actin*, *hprt1*, and *ppia*) that we tested in an earlier bunting study^57^. We then calculated the ΔCT value by subtracting threshold cycle (Ct) of the reference gene from the target gene [Ct(target) – Ct(reference)], and then ΔCt was normalized against ΔCt of a calibrator sample, which was composed of a mixture of cDNA from both time points (hour 10 and 18); this gave the ΔΔCt value, which was plotted as a negative value powered to two (2^−ΔΔCt^)^37,38,57^. Unpaired Student’s t-test tested differences in mRNA expression between two groups.

### Transcriptome-wide assays

#### RNA extraction, library preparation and sequencing

The RNA-Seq was performed for a total of eight hypothalamus samples from each state covering all the four-time points (hours 10, 14, 18 and 22) during the 22 h of the light exposure (n = 2/time point/state). The total RNA was extracted by using TRIzol plus RNA purification kit (12183555, Thermofisher Scientific, Wilmington, DE, USA). The RNA quality check, library preparation and sequencing were done commercially. From this, RNA libraries were prepared from ~ 1 μg of total RNA by using Illumina TruSeq Stranded total RNA library preparation kit, as per the manufacturer’s protocol. Briefly, the total RNA was Ribo depleted using plant rRNA removal mix and rRNA removal beads, and then subjected to the purification, fragmentation and priming for cDNA synthesis. Ribo-depleted and fragmented RNA was converted into first-strand cDNA, followed by the second-strand cDNA synthesis, A-tailing, adapter-index ligation, and finally amplified by recommended PCR cycles. Both quality and quantity checks of the library were done by using Agilent DNA High Sensitivity Assay Kit. One hypothalamus sample from hour 22 of the photorefractory state did not pass the quality check; so it was excluded from further analysis (hence, only one hypothalamus sample represents the hour 22 in photorefractory state). The Bioanalyzer 2100 (Agilent Technologies) analyzed amplified libraries using a High Sensitivity DNA chip, as per the manufacturer's instructions. After obtaining Qubit concentration for both library and mean peak size from Bioanalyzer profile, the library was loaded on to the Illumina HiSeq 2500 platform, and sequenced as 150-bp paired end reads.

#### Transcriptome assembly and annotation

Figure 1B illustrates the workflow for the transcriptome assembly and annotation. For primary processing, we performed the adaptor trimming (clipping of the adapter sequences) and quality check (dropping the reads with quality score < 30) using Trimmomatic (version 0.36, RWTH Aachen University, Germany), and this followed by sequence assembly using trinityrnaseq (version 2.2.0). The trinity.fasta files were processed using CD-HIT, which corrects the biasness in sequence files and reduces the sequence redundancy. The sequences were then pseudo-aligned by Kallisto, that uses a de Bruijn graph to “pseudo-align” reads to an index transcriptome^62^. Briefly, this procedure included construction of an index file using sequence files from all time points of both photosensitive and photorefractory states. The reads were then pseudo-aligned to the index file and quantified in transcripts per million (TPM) units. The transcripts with effective count ≥ 5 were used for further analysis. Because of the low sample size (n = 2/time point/state), TPMs were compared pairwise in a reduced matrix design, and differentially expressed genes (DEGs) were identified using R Bioconductor package edgeR and limma with default parameters at *p*_*adj.*_ ≤ 0.05^63^. The results obtained from differential gene expressions were summarized in a volcano plot, which is essentially a scatter plot based on the magnitude of change (fold change) with respect to statistical significance (*p*_*adj*_ value). A volcano plot helps visualization of the overall changes in gene expressions in the entire data set. An accelerated BlastX program (Diamond, version 0.9.21) was used to annotate the gene sequences against the non-redundant (nr) database.

#### Pathway enrichment and network analysis of DEGs

For functional annotation and to show a functional linkage of the DEGs both within the state and between states, we performed STRING analysis (stringdb.org, version 11.0). This network analysis predicts protein–protein interaction from the provided gene list. In this analysis, gene names are mapped to those of the reference species, for which we used a closely related migratory collared flycatcher (*Ficedula albicollis*); the proteins encoded by these genes are identified. The mapped proteins were then functionally annotated based on KEGG data^64^, Pfam and Uniprot on the STRING platform itself. The enrichment is based on the number of genes observed vs. the number of known genes in a particular pathway. *padj.* ≤ 0.05 indicated a significantly enriched pathway. The identified proteins were then used to draw an interaction network based on confidence values; *i.e.* the strength of data support based on the text mining, experiments, databases, co-expression and co-occurrence. Here, the minimum interaction score was set to high confidence of 0.700. Based on this, the nodes that formed a network were presented in the STRING diagram; nodes represent the proteins, and edges represent the protein–protein interaction.

### Statistics

Statistical analysis for comparison of data on mRNA expression levels from blood and hypothalamus tissues, obtained by qPCR assays, was performed using the Graph Pad Prism software (version 6.0, San Diego, CA, USA). For statistical significance, alpha was set at 0.05.

## Supplementary Information

Supplementary Information.

## Data Availability

The RNA-Seq data have been deposited to Gene Expression Omnibus (GEO) database hypothalamus: GSE165319). cDNA sequences used for qPCR can be accessed using the accession numbers, as listed in the Table S1.
