# Supplementary material for: Photoperiodically driven transcriptome-wide changes in the hypothalamus reveal transcriptional differences between physiologically contrasting seasonal life-history states in migratory songbirds
Source: Sci Rep. 2021 Jun 17;11:12823. doi: 10.1038/s41598-021-91951-4 (PMC8211672; doi:10.1038/s41598-021-91951-4)
Supplement: Supplementary file 1 — Supplementary Information. [file 41598_2021_91951_MOESM1_ESM.pdf]

Photoperiodically driven transcriptome-wide changes in the hypothalamus reveal transcriptional differences between crucial life-history states in migratory songbirds

Aakansha Sharma[1], Subhajit Das[1], Sayantan Sur[1], Jyoti Tiwari[2], Khushboo Chaturvedi[2], Neha Agarwal[1], Shalie Malik[2], Sangeeta Rani[2] and Vinod Kumar[1#]

[1]Department of Zoology, University of Delhi, Delhi 110007, India; [2]Department of Zoology, University of Lucknow, Lucknow, 226007, India

Running title: LHS-dependent transcriptional response

[#]Corresponding author: Email: drvkumar11@yahoo.com

Keywords: Bunting; Gene expression; Hypothalamus; Photoperiod; Transcriptome; Life-history state

Supplementary Information

Table S1: Gene specific primers used for measurement of mRNA expression by q-PCR

Gene	Primer Sequence	Accession No.
ba	F: 5’ TGTTACCCACACTGTGCCCATCTA 3’<br>R: 5’ TTCATGAGGTAGTCCGTCAGGTCA 3’	KC874663.1
prdx4	F: 5’ ATGAACGACCTTCCTGTTGG 3’<br>R: 5’ AGGGCAAACTTCTCCATGTT 3’	MH019229
gpx1	F: 5’ CCTCTGTTCACCTTCCTGAAA 3’<br>R: 5’ AGATGATGTACTGCGGGTTG 3’	MK868464
sod1	F: 5’ TGCGGAGTGATTGGGATTG 3’<br>R: 5’ CATCTGGAAGTTGCAGGAAGA 3’	MK868466
il1β	F: 5’ CTGCTTCAGGAGCTTGGT 3’<br>R: 5’ CATCCAGGTGACAGTGACAA 3’	MK868470
tsh β	F: 5’ TTCTGCATGACTCGGGACAGCAAT 3’<br>R: 5’ TGTATGTGCACACGTTCTGGGACA 3’	KC404637
dio2	F: 5’ CAAGTGTGGAACCAAATGCCACCT 3’<br>R: 5’ TAGCTGAGCCAAAGTTGACCACCA 3’	KJ624635
dio3	F: 5’ CGTGGACACCATGGACAATGCTT 3’<br>R: 5’ TCACCTTCTCCTCCTGGATGATGT 3’	KJ624636
gnrh	F: 5’ CCAGGAGGTTGCAAATGAAATG 3’<br>R: 5’ CTTGTTCTTCCATCAGCCTCTC 3’	KJ624637

Table S2: Mean (± SD) raw reads, pseudoaligned reads and transcripts with non-zero abundance from hypothalamus samples. (Note: hour 22 in photorefractory state – n =1)

**Sample ID (State/ hour)**	**Raw reads (number)**	**Pseudoaligned reads (number)**	**Transcript with non-zero abundance (number)**	**Correlation coefficient (R^2)**
photosensitive/ hour 10	9,999,979 ± 2,362,601	6,473,672 ± 1,194,713	71,068 ± 2,309	0.740
photosensitive/ hour 14	8,424,733 ± 408973	5,518,765 ± 297,014	60,815 ± 1,295	0.871
photosensitive/ hour 18	7,699,760 ± 32209	4,941,345 ± 185,868	52,759 ± 6,971	0.935
photosensitive/ hour 22	12,401,649 ± 1,115,769.	8,736,432 ± 943,975	85,058 ± 4,233	0.942
photorefractory/ hour 10	7,649,673 ± 154,470	4,984,562 ± 959,437	52,289 ± 2,553	0.912
photorefractory/ hour 14	9,697,773 ± 1,974,230	6,343,760 ± 1,053,815	62,977 ± 2,218	0.924
photorefractory/ hour 18	4,986,590 ± 343,856	3,308,757 ± 187,658	40,635 ± 1,173	0.826
photorefractory/ hour 22	12,892,408	8,951,449	87,525	-

Table S3: Differentially expressed ($p_{adj.} < 0.05$) hypothalamic genes within and between photosensitive and photorefractory life history states of blackheaded buntings on the first day of long day

a) Photosensitive (hour 10 vs. hour 14)

gene symbol	description	log2 fold change	p-value	$p_{adj.}$
ttr	transthyretin	-5.357	4.94E-10	8.0E-06
atp6v1e1	V-type proton ATPase subunit E1	5.704	4.78E-09	3.6E-05
pomc	pro-opiomelanocortin	-4.771	7.19E-08	0.0004
trim28	transcription intermediary factor 1-beta, partial	4.850	9.46E-08	0.0005
pgam1	phosphoglycerate mutase 1	-5.490	3.81E-06	0.010
tppp3	tubulin polymerization-promoting protein family member 3	-5.403	6.00E-06	0.015
mbp	myelin basic protein isoform X1	3.858	8.78E-06	0.021
uqcrc1	cytochrome b-c1 complex subunit 1, mitochondrial	-8.085	9.48E-06	0.021
atp6v1b2	V-type proton ATPase subunit B, brain isoform	-5.141	2.25E-05	0.039
lyz	lysozyme C	7.872	2.82E-05	0.044

b) Photosensitive (hour 10 vs. hour 18)

gene symbol	description	log2 fold change	p-value	$p_{adj.}$
ttr	transthyretin	-8.280	1.2E-13	5.4E-09
atp6v1e1	V-type proton ATPase subunit E1	6.147	4.2E-10	4.8E-06
pomc	pro-opiomelanocortin	-5.641	4.8E-09	3.3E-05
eno1	alpha-enolase	3.613	2.4E-06	0.007
apo a-i	apolipoprotein A-I	3.415	2.5E-06	0.007
ifi27l2b	interferon alpha-inducible protein 27-like protein 2B	-3.630	2.9E-06	0.007
cct8	T-complex protein 1 subunit theta isoform X2	-7.855	2.8E-05	0.047
pcca	propionyl-CoA carboxylase alpha chain, mitochondrial	3.315	3.6E-05	0.049

c) Photosensitive (hour 10 vs. hour 22)

gene symbol	description	log2 fold change	p-value	$p_{adj.}$
nsf	vesicle-fusing ATPase	9.917	3.2E-10	4.2E-06
pomc	pro-opiomelanocortin	-4.960	1.7E-08	7.5E-05
atp6v1e1	V-type proton ATPase subunit E 1	5.366	2.1E-08	8.5E-05
ifi27l2b	interferon alpha-inducible protein 27-like protein 2B	-4.067	3.2E-07	0.0007
b2m	beta-2-microglobulin	-4.218	6.6E-07	0.0012
slc6a8	sodium- and chloride-dependent creatine transporter 1	-8.452	1.7E-06	0.002

tnfrsf21	tumor necrosis factor receptor superfamily member 21 isoform X2	4.855	2.7E-06	0.003
clu	clusterin	-3.956	3.9E-06	0.004
loc107604320	chitin-binding lectin 1-like	-3.608	5.4E-06	0.005
banf1	barrier-to-autointegration factor-like, partial	-3.935	6.4E-06	0.006
plp1	myelin proteolipid protein isoform X1	-3.676	7.1E-06	0.007
phf24	PHD finger protein 24 isoform X2	4.126	8.0E-06	0.007
hax1	HCLS1-associated protein X-1 isoform X1	-3.751	1.2E-05	0.010
pde2a	cGMP-dependent 3',5'-cyclic phosphodiesterase isoform X1	5.028	2.7E-05	0.017
wbp1	WW domain-binding protein 1	-3.905	3.0E-05	0.018
loc101806991	protein lifeguard 1-like	-3.107	3.1E-05	0.019
agbl5	cytosolic carboxypeptidase-like protein 5, partial	-3.912	6.0E-05	0.031
hnrnph1	heterogeneous nuclear ribonucleoprotein H isoform X5	-4.408	6.8E-05	0.035
ndufb5	NADH dehydrogenase [ubiquinone] 1 beta subcomplex subunit 5, mitochondrial isoform X1	-5.000	6.9E-05	0.035
cga	glycoprotein hormones alpha chain	-4.372	8.0E-05	0.039
elmod1	ELMO domain-containing protein 1	-7.621	9.6E-05	0.044
oc101810511	uncharacterized protein	3.160	0.0001	0.049
cadps	calcium-dependent secretion activator 1	-7.557	0.0001	0.049

d) Photorefractory (hour 10 vs. hour 14)

gene symbol	description	log2 fold change	p-value	$p_{adj.}$
ttr	transthyretin	-6.358	1.2E-12	5.2E-08
hsp90aa1	heat shock protein HSP 90-alpha	6.719	3.6E-09	3.3E-05
eno1	alpha-enolase	6.034	4.5E-09	3.3E-05
ha1f	class I histocompatibility antigen, F10 alpha chain-like isoform X1	-4.688	7.9E-08	0.0004
eef1a1	elongation factor 1-alpha 1	4.610	8.4E-07	0.0026
eif4g2	eukaryotic translation initiation factor 4 gamma 2, partial	-8.185	6.3E-06	0.014
apo a-i	apolipoprotein A-I	3.066	1.7E-05	0.030
pdyn	proenkephalin-B isoform X2	-7.923	2.2E-05	0.037
arhgdig	rho GDP-dissociation inhibitor 3	7.903	2.2E-05	0.037
ifi27l2b	interferon alpha-inducible protein 27-like protein 2B	-3.038	4.0E-05	0.042

e) Photorefractory (hour 10 vs. 18)

gene symbol	description	log2 fold change	p-value	$p_{adj.}$
pank4	pantothenate kinase 4	7.495	1.5E-11	5.02E-07
ppp1r1b	protein phosphatase 1 regulatory subunit 1B	-5.032	2.7E-10	0.000002
eno1	alpha-enolase	6.362	3.0E-10	0.000002

loc101818738	probable glutamate receptor	5.585	3.7E-08	0.00010
eef1a1	elongation factor 1-alpha	4.899	2.2E-07	0.0003
tomm6	mitochondrial import receptor subunit TOM6 homolog	-4.474	2.4E-07	0.0004
uqcrc1	cytochrome b-c1 complex subunit 1, mitochondrial	-5.909	8.4E-07	0.001
wbp1	WW domain-binding protein 1	-4.112	2.0E-06	0.002
dctn1	dynactin subunit 1	-4.084	2.3E-06	0.002
dstn	destrin	3.692	1.4E-05	0.009
atp5mc1	ATP synthase F(0) complex subunit C1, mitochondrial	-3.189	1.6E-05	0.010
rgcc	regulator of cell cycle RGCC	4.110	2.2E-05	0.0132
agbl5	cytosolic carboxypeptidase-like protein 5, partial	-3.567	2.7E-05	0.015
loc101813953	neuropeptide-like protein C4orf48 homolog isoform X1	-3.517	2.9E-05	0.016
pfkl	ATP-dependent 6-phosphofructokinase, liver type	-3.732	3.3E-05	0.016
sema6b	semaphorin-6B	-3.513	3.8E-05	0.018
hsp90aa1	heat shock protein HSP 90-alpha	-3.318	3.9E-05	0.019
tk2	thymidine kinase 2, mitochondrial	4.902	4.7E-05	0.021
kcnj8	ATP-sensitive inward rectifier potassium channel 8	-4.141	5.6E-05	0.024
atp6v1h	V-type proton ATPase subunit H	4.787	8.4E-05	0.033
mbp	myelin basic protein isoform X1	2.770	9.2E-05	0.035
nap1l1	nucleosome assembly protein 1-like 1 isoform X1	4.705	0.0001	0.038
cox14	cytochrome c oxidase assembly protein COX14 isoform X1	-2.759	0.0001	0.038
npas2	neuronal PAS domain-containing protein 2	-4.271	0.0001	0.044
spag7	sperm-associated antigen 7	-3.087	0.0001	0.045
tmem61	transmembrane protein 61 isoform X1	-3.318	0.0002	0.050
aldh2	aldehyde dehydrogenase, mitochondrial	4.572	0.0002	0.050

f) Photorefractory (hour 10 vs. hour 22)

gene symbol	description	log2 fold change	p-value	$p_{adj.}$
eef1a1	elongation factor 1-alpha 1	5.286	1.04E-08	3.2E-05
pcca	propionyl-CoA carboxylase alpha chain, mitochondrial	-5.066	3.87E-08	0.00009
olah	S-acyl fatty acid synthase thioesterase, medium chain	-6.201	1.18E-07	0.0001
nr1d1	nuclear receptor subfamily 1 group D member 1	5.278	2.64E-07	0.0003
atp5mf	ATP synthase subunit f, mitochondrial	-3.743	8.70E-07	0.0008
loc101818738	probable glutamate receptor	5.075	8.84E-07	0.0008
syt11	synaptotagmin-11 isoform X1	4.110	2.31E-06	0.0019
agbl5	cytosolic carboxypeptidase-like protein 5, partial	-4.149	7.66E-06	0.0046

ppp1r1b	protein phosphatase 1 regulatory subunit 1B	-3.232	7.84E-06	0.0046
ak5	adenylate kinase isoenzyme 5	-4.254	8.88E-06	0.0051
map1a	microtubule-associated protein 1A	4.236	1.34E-05	0.0070
arhgdig	rho GDP-dissociation inhibitor 3	8.021	1.45E-05	0.0074
taf9b	transcription initiation factor TFIID subunit 9B	3.417	1.91E-05	0.009
ha1f	class I histocompatibility antigen, F10 alpha chain-like isoform X1	-3.318	2.03E-05	0.009
pgap3	post-GPI attachment to proteins factor 3 isoform X1	-4.177	3.70E-05	0.015
ndrg4	protein NDRG4 isoform X4	2.958	5.77E-05	0.021
loc101813953	neuropeptide-like protein C4orf48 homolog isoform X1	-3.285	6.12E-05	0.021
loc101810511	uncharacterized protein	3.154	6.62E-05	0.023
atp5f1b	ATP synthase subunit beta, mitochondrial, partial	2.915	7.30E-05	0.024
phgdh	D-3-phosphoglycerate dehydrogenase	-7.668	7.45E-05	0.024
snca	alpha-synuclein	-2.822	8.56E-05	0.027
pitpnc1	cytoplasmic phosphatidylinositol transfer protein 1-like	-3.650	9.81E-05	0.031
cd151	CD151 antigen	3.646	0.0001	0.031
ctsd	cathepsin D	4.783	0.0001	0.031
pank4	pantothenate kinase 4	4.783	0.0001	0.031
sept4	septin-4	3.800	0.0001	0.033
tac1	protachykinin-1 isoform X2	-3.932	0.0001	0.036
elmod1	ELMO domain-containing protein 1	3.541	0.0001	0.039
mbp	myelin basic protein isoform X1	2.652	0.0002	0.043
rpl19	60S ribosomal protein L19, partial	-3.110	0.0002	0.044
tpd52l1	tumor protein D53 isoform X1	-3.863	0.0002	0.045
cadps	calcium-dependent secretion activator 1	-4.149	0.0002	0.047
pfkl	ATP-dependent 6-phosphofructokinase, liver type	3.065	0.0002	0.048
wbp1	WW domain-binding protein 1	-2.945	0.0002	0.048
aldoa	fructose-bisphosphate aldolase A-like	3.241	0.0002	0.049
gpm6b	neuronal membrane glycoprotein M6-b isoform X2	3.457	0.0002	0.049
slc6a8	sodium- and chloride-dependent creatine transporter 1	-4.732	0.0002	0.050
ndufa13	NADH dehydrogenase [ubiquinone] 1 alpha subcomplex subunit 13	-2.683	0.0002	0.050
banf1	barrier-to-auto integration factor-like, partial	-3.343	0.0003	0.050

g) Photosensitive hour 10 vs. photorefractory hour 10

gene symbol	description	log2 fold change	p-value	$p_{adj.}$
pomc	pro-opiomelanocortin	6.297	8.8E-10	1.0E-05

atp6v1e1	V-type proton ATPase subunit E 1	-6.860	1.6E-09	1.5E-05
hsp90aa1	heat shock protein HSP 90-alpha	5.903	4.0E-07	0.0016
loc101821968	class I histocompatibility antigen, F10 alpha chain-like isoform X2	-4.179	5.8E-07	0.0021
pank4	pantothenate kinase 4	5.517	3.3E-06	0.0100
loc101818738	probable glutamate receptor	4.852	3.9E-06	0.0106
pcca	propionyl-CoA carboxylase alpha chain	-3.695	5.9E-06	0.0134
aldh2	aldehyde dehydrogenase, mitochondrial	-5.100	3.2E-05	0.04
eif4g2	eukaryotic translation initiation factor 4 gamma 2, partial	-5.022	4.7E-05	0.049

h) Photosensitive hour 14 vs. photorefractory hour 14

gene symbol	description	log2 fold change	p-value	$p_{adj.}$
atp5md	up-regulated during skeletal muscle growth protein 5	4.918	6.0E-08	0.0006
tppp3	tubulin polymerization-promoting protein family member 3	-5.459	5.1E-06	0.018
hcrt	orexin	5.409	6.0E-06	0.018
uqcrc1	cytochrome b-c1 complex subunit 1, mitochondrial	-7.965	1.8E-05	0.039
eef1a1	elongation factor 1-alpha 1	-3.662	1.8E-05	0.039

i) Photosensitive hour 18 vs. photorefractory hour 18

gene symbol	description	log2 fold change	p-value	$p_{adj.}$
ttr	transthyretin	-10.213	1.4E-19	5.0E-15
eef1a1	elongation factor 1-alpha 1	-6.701	1.8E-09	0.000008
pank4	pantothenate kinase 4	-4.377	1.6E-07	0.0003
dctn1	dynactin subunit 1	4.415	4.3E-07	0.0006
ifi27l2b	interferon alpha-inducible protein 27-like protein 2B	-3.848	8.4E-07	0.001
tomm6	mitochondrial import receptor subunit TOM6 homolog	4.185	1.6E-06	0.002
haus4	HAUS augmin-like complex subunit 4, partial	4.625	1.7E-06	0.002
synpr	synaptoporin isoform X1	5.749	2.5E-06	0.002
scd5	stearoyl-CoA desaturase 5, partial	-3.833	5.4E-06	0.004
cart	cocaine- and amphetamine-regulated transcript protein	4.399	5.8E-06	0.004
agbl5	cytosolic carboxypeptidase-like protein 5, partial	3.771	1.2E-05	0.007
wbp1	WW domain-binding protein 1	3.825	1.2E-05	0.007
loc101811864	corticoliberin-like	3.982	1.2E-05	0.007
atp5mc1	ATP synthase F(0) complex subunit C1, mitochondrial	3.201	1.5E-05	0.009
atp5md	up-regulated during skeletal muscle growth protein 5	3.175	1.8E-05	0.009
cct8	T-complex protein 1 subunit theta isoform X2	-7.943	1.8E-05	0.009

apo a-i	apolipoprotein A-I	2.981	2.6E-05	0.013
cox14	cytochrome c oxidase assembly protein COX14 isoform X1	2.924	4.2E-05	0.018
spag7	sperm-associated antigen 7	3.248	5.1E-05	0.021
uqcrc1	cytochrome b-c1 complex subunit 1, mitochondrial	5.136	5.7E-05	0.023
grp	gastrin-releasing peptide, partial	3.958	5.8E-05	0.023
pold4	DNA polymerase delta subunit 4	3.127	6.6E-05	0.025
coa6	cytochrome c oxidase assembly factor 6 homolog	2.971	6.7E-05	0.025
prdx1	peroxiredoxin-1	4.435	8.0E-05	0.029
loc101817208	neurophysin 1-like isoform X2	3.061	9.5E-05	0.034
npas2	neuronal PAS domain-containing protein 2	4.396	9.5E-05	0.034
timm10b	mitochondrial import inner membrane translocase subunit Tim10 B	3.802	0.0001	0.037
tubb3	tubulin beta-3 chain	2.687	0.0002	0.043
atp6v1c1	V-type proton ATPase subunit C 1	-3.803	0.0002	0.048
klc1	kinesin light chain 1 isoform X6	3.420	0.0002	0.049
oas1	2'-5'-oligoadenylate synthase 1-like	-3.018	0.0002	0.050
tk2	thymidine kinase 2, mitochondrial	3.348	0.0002	0.050
sncg	gamma-synuclein	2.601	0.0002	0.050

j) Photosensitive hour 22 vs. photorefractory hour 22

**gene symbol**	**description**	**log2 fold change**	**p-value**	$\mathbf{p}_{adj.}$
stxbp1	syntaxin-binding protein 1 isoform X2	-9.270	1.6E-08	0.0001
eno1	alpha-enolase	4.826	4.4E-08	0.0002
rpl37	60S ribosomal protein L37	-4.615	6.7E-08	0.0003
rpl12	60S ribosomal protein L12	-4.797	1.5E-07	0.0005
elmod1	ELMO domain-containing protein 1	-8.770	3.0E-07	0.0009
hsp90aa1	heat shock protein HSP 90-alpha	5.715	1.1E-06	0.002
trim9	E3 ubiquitin-protein ligase TRIM9	-4.848	5.8E-06	0.011
ifi27l2b	interferon alpha-inducible protein 27-like protein 2B	-3.442	8.9E-06	0.016
loc101808646	avidin-like	-4.691	1.3E-05	0.021
mapre3	microtubule-associated protein RP/EB family member 3 isoform X1	4.542	1.7E-05	0.025
sod1	superoxide dismutase [Cu-Zn]	-3.309	1.7E-05	0.025
pde2a	cGMP-dependent 3',5'-cyclic phosphodiesterase isoform X1	5.120	2.3E-05	0.029
tnfrsf21	tumor necrosis factor receptor superfamily member 21 isoform X2	4.485	2.3E-05	0.029
atp6v1b2	V-type proton ATPase subunit B, brain isoform	5.048	3.2E-05	0.037
kcnab2	voltage-gated potassium channel subunit beta-2 isoform X5	7.826	3.6E-05	0.041

Table S4: List of common differentially expressed hypothalamic genes within and between photosensitive and photorefractory life history states of blackheaded buntings on the first day of long day

A. Within state comparison

Within photosensitive state (cf. Figure 2d)			
**hour 10 vs. 14 ∩ hour 10 vs. 18**	**hour 10 vs. 14 ∩ hour 10 vs. 22**	**hour 10 vs. 18 ∩ hour 10 vs. 22**	**hour 10 vs. 14 ∩ hour 10 vs. 18 ∩ hour 10 vs. 22**
ttr	*atp6v1e1*	*atp6v1e1*	*atp6v1e1*
atp6v1e1	*pomc*	*pomc*	*pomc*
pomc		*ifi27l2b*	
**Within photorefractory state (cf. Figure 2h)**			
**hour 10 vs. 14 ∩ hour 10 vs. 18**	**hour 10 vs. 14 ∩ hour 10 vs. 22**	**hour 10 vs. 18 ∩ hour 10 vs. 22**	**hour 10 vs. 14 ∩ hour 10 vs. 18 ∩ hour 10. vs. 22**
hsp90aa1	*ha1f*	*pank4*	*eef1a1*
eno1	*eef1a1*	*ppp1r1b*	
eef1a1	*arhgdig*	*loc101818738*	
		eef1a1	
		wbp1	
		agbl5	
		loc101813953	
		pfkl	
		mbp	

B. Between state comparison

**photosensitive vs. photorefractory state (cf. Figure 2m)**		
**hour 10 ∩ hour 22**	**hour 14 ∩ hour 18**	**hour 18 ∩ hour 22**
hsp90aa1	*atp5md*	*ifi27l2b*
	uqcrc1	
	eef1a1	
**hour 10 ∩ hour 14** = 0 (No common genes)		
**hour 10 ∩ hour 14 ∩ hour 18** = 0		
**hour 10 ∩ hour 14 ∩ hour 22** = 0		
**hour 10 ∩ hour 18 ∩ hour 22** = 0		
**hour 14 ∩ hour 18 ∩ hour 22** = 0		
**hour 10 ∩ Hour 14 ∩ hour 18 ∩ hour 22** = 0		
